# Structural and Functional Characterization of the Human Thymidylate Synthase (hTS) Interface Variant R175C, New Perspectives for the Development of hTS Inhibitors

**DOI:** 10.3390/molecules24071362

**Published:** 2019-04-07

**Authors:** Cecilia Pozzi, Stefania Ferrari, Rosaria Luciani, Maria Paola Costi, Stefano Mangani

**Affiliations:** 1 Department of Biotechnology, Chemistry and Pharmacy, Department of Excellence 2018-2020, University of Siena, via Aldo Moro 2, 53100 Siena, Italy; 2 Department of Life Sciences, University of Modena and Reggio Emilia, Via Campi 103, 41125 Modena, Italy; sferrari591@gmail.com (S.F.); rosaria.luciani@gmail.com (R.L.); mariapaola.costi@unimore.it (M.P.C.)

**Keywords:** human thymidylate synthase, interface variant, active conformation, tethering approach, X-ray crystallography, anticancer drugs

## Abstract

Human thymidylate synthase (hTS) is pivotal for cell survival and proliferation, indeed it provides the only synthetic source of dTMP, required for DNA biosynthesis. hTS represents a validated target for anticancer chemotherapy. However, active site-targeting drugs towards hTS have limitations connected to the onset of resistance. Thus, new strategies have to be applied to effectively target hTS without inducing resistance in cancer cells. Here, we report the generation and the functional and structural characterization of a new hTS interface variant in which Arg175 is replaced by a cysteine. Arg175 is located at the interface of the hTS obligate homodimer and protrudes inside the active site of the partner subunit, in which it provides a fundamental contribution for substrate binding. Indeed, the R175C variant results catalytically inactive. The introduction of a cysteine at the dimer interface is functional for development of new hTS inhibitors through innovative strategies, such as the tethering approach. Structural analysis, performed through X-ray crystallography, has revealed that a cofactor derivative is entrapped inside the catalytic cavity of the hTS R175C variant. The peculiar binding mode of the cofactor analogue suggests new clues exploitable for the design of new hTS inhibitors.

## 1. Introduction

Thymidylate synthase (TS, EC 2.1.1.45) is a ubiquitous enzyme that catalyses the reductive methylation of 2’-deoxyuridine-5’-monophosphate (dUMP) to 2’-deoxythymidyne-5’-monophosphate (dTMP) using *N*^5^,*N*^10^-methylenetetrahydrofolate (mTHF) as cofactor. In human cells, TS provides the only synthetic source of dTMP necessary for DNA biosynthesis. As a matter of fact, its inhibition halts the replication processes and induces apoptosis in rapidly dividing cells, an effect known as “thymineless death” [1]. Given its pivotal role for cell survival and proliferation, human TS (hTS) represents a validated target in anticancer chemotherapy. Nonetheless, treatment with classical hTS inhibitors, directed to the enzyme active site, such as FdUMP (the active metabolite of 5-fluorouracil) and raltitrexed, has limitations due to the onset of resistance mechanisms relying on TS overexpression [2,3]. Thus, other strategies have to be applied to effectively target hTS without inducing resistance in cancer cells. 

**Figure 1:**
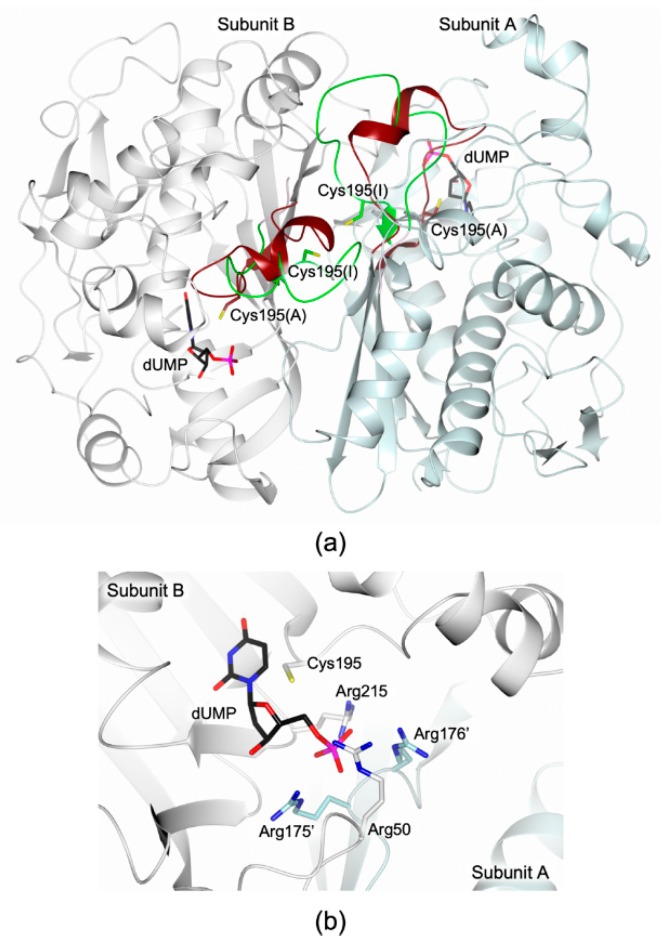
(**a**) Cartoon representation of the superimposition between the hTS homodimer (subunits A and B are coloured light cyan and white, respectively) in the active (PDB id 5X5D [11]) and inactive (PDB id 3N5G [12]) conformations. The two orientations of the catalytic loop (residues 181–197), defining the active (brown trance) and inactive (green trace) conformations, are displayed. The catalytic cysteine is shown in sticks in the active (A) and inactive (I) conformations (brown and green carbons, respectively). The position of the catalytic cavity is indicated by the presence of the substrate 2’-deoxyuridine-5’-monophosphate (dUMP, in sticks, black carbons; PDB id 5X5D [11]). (**b**) Four-arginine cluster anchoring the dUMP phosphate in the hTS active site. The cluster is composed by Arg50, Arg215, Arg175’, and Arg176’ (the last two from the partner subunit). In all figures, nitrogen atoms are coloured blue, oxygen red, sulphur yellow, and phosphorous magenta.

hTS is an obligate homodimer (Figure 1a) with residues from both subunits that contribute to create the substrate binding site. Notably, two arginine residues, Arg175’ and Arg176’, from the cognate subunit protrude into the active site contributing to form the four-arginine cluster that anchor the substrate, together with Arg50 and Arg215 (Figure 1b). Former structural analysis on hTS has revealed that the enzyme homodimers can switch between the active and the inactive conformation, primarily differing in the orientation of the catalytic loop (residues 181–197) (Figure 1a) [4,5]. In the active conformation the catalytic Cys195 is exposed inside the catalytic cavity whereas, in the inactive conformation of the enzyme, it is moved at the dimer interface. The transition to the active conformation is fundamental to create a functional active site in which dUMP can bind followed by the cofactor [5]. 

Further its catalytic activity, hTS plays also the role of regulatory protein by binding RNAs, including its own mRNA (TSmRNA) [2,3]. It has been proposed that inhibitor binding to the enzyme active site reduces the affinity of hTS for the TSmRNA, removing the translational arrest and triggering TS overexpression responsible for drug-induced resistance [3]. Despite the TSmRNA binding site on hTS being still uncharacterized, there is evidence that the dimer interface plays an important role in hTS-mRNA recognition, perhaps by controlling protein conformational changes that alternatively expose and hide the TSmRNA recognition site [6,7,8]. 

In the present work, we report on the generation, expression, purification, and characterization of the hTS variant R175C. In hTS, residue 175 is exposed at the dimer interface, an area that is critical for the enzyme function and dimerization [9]. The introduction of a cysteine residue at the dimer interface is potentially exploitable as anchoring point for new drug-discovery strategies based on the tethering approach [10]. The replacement of Arg175 with a cysteine results in the complete loss of enzyme activity, proving that Arg175 is fundamental to anchor the substrate inside the catalytic cavity. The determination of the X-ray crystal structure of hTS R175C reveals that this variant adopts the active conformation. Furthermore, a cofactor analogue molecule populates the catalytic cavity of only one enzyme subunit with a peculiar binding mode, providing clues exploitable for the design of new hTS inhibitors. 

## 2. Results and Discussion

### 2.1. Variant Production and Characterization

The hTS variant R175C was generated through site-directed mutagenesis, using the gene coding sequence for the wild-type enzyme as template for the PCR reaction (see the Materials and Methods section). The resulting expression plasmid hTS-R175C-pQE80L encodes also for a non-cleavable *N*-terminal His^6^-tag. The variant was expressed as His^6^-tag protein (HT-hTS R175C) in the bacterial strain *E. coli* BL21(DE3), in which it is mainly localized in the soluble cellular fraction (analogously to the wild-type enzyme [9,13]). The purification procedure took advantage from the introduction of the *N*-terminal His^6^-tag, indeed an almost pure (>95%) protein sample was obtained after the first purification step relying on nickel-affinity chromatography. The purification was completed through size exclusion chromatography, resulting in a highly pure protein sample (>98%). The HT-hTS R175C elution profile was consistent with the enzyme dimer assembly (Figure S1). The final production yield was estimated to ~150 mg L^−1^, comparable to that of the wild-type enzyme expressed and purified under the same conditions [9,13]. Dynamic light scattering (DLS) was performed on HT-hTS R175C, resulting in a protein hydrodynamic radius of approximately 3.8 nm, consistent with values formerly reported for the wild-type enzyme [14]. The hydrodynamic radius of both HT-hTS R175C and the wild-type enzyme are indicative of the dimeric quaternary assembly (MW-R of 75–78 kDa) [14].

The enzymatic activity assays, performed on HT-hTS R175C, showed that the mutant is catalytically inactive (no detectable activity was observed during the assays). This result is in agreement with former studies on a further hTS variant bearing an alanine in the same position (hTS variant R175A), for which the same inactive catalytic profile was reported [9]. The mutation of Arg175, belonging to the four-arginine cluster that anchors the substrate phosphate moiety in the TS active site (Figure 1b), has a dramatic effect on the enzyme activity. Reasonably, the loss of this arginine impairs/alters the correct positioning of the substrate in the active site, as formerly suggested for the R175A variant [9]. 

### 2.2. Structrural Characterization 

**Figure 2:**
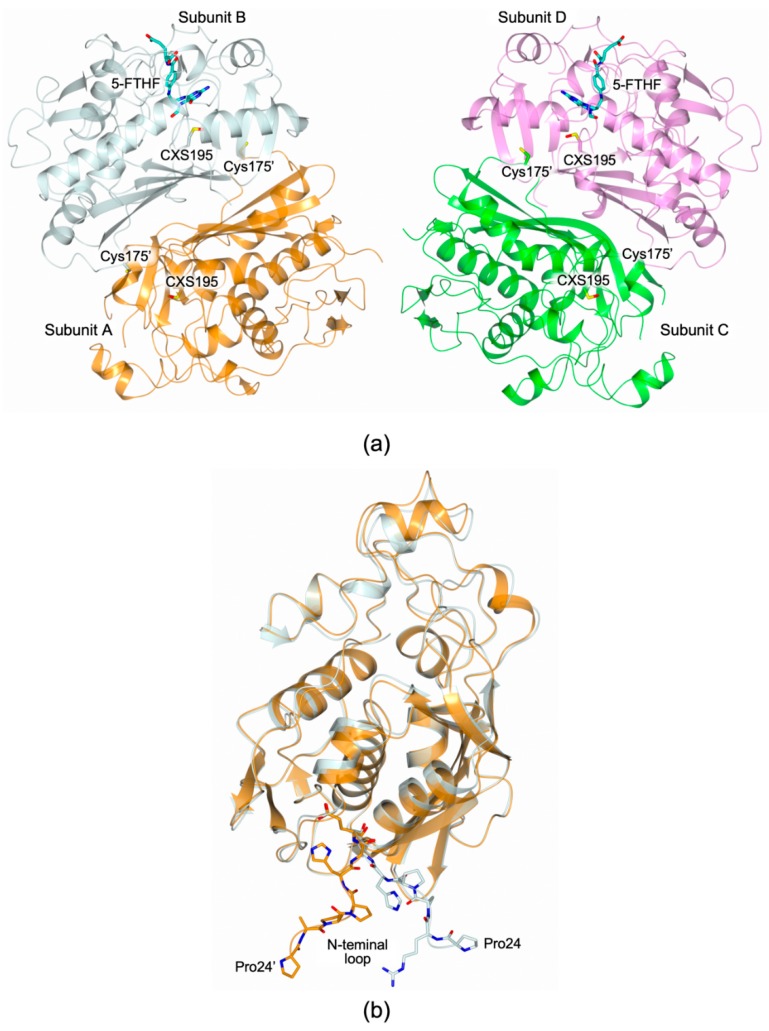
(**a**) Cartoon representation of the two homodimers (A-B and C-D, in our model) found in the cell ASU (subunit A is coloured orange, B light cyan, C green and D pink) of HT-hTS R175C. Within each enzyme homodimer, both subunits assume the active conformation, showing the catalytic Cys195 exposed inside the active site. In all subunits, the catalytic Cys195 is modified as S-oxy-cysteine (CSX195, in sticks, carbon atoms are color-coded according to the parent subunit). The mutated residue Cys175 is displayed in sticks (carbon atoms are color-coded according to the parent subunit). Both enzyme dimers display asymmetrical ligand binding within their active sites, indeed the cofactor analogue 5-formyl-6-tetrahydrofolate (5-FTHF, in sticks, cyan carbons) is entrapped in the active site of subunit B and D. (**b**) The structural comparison between subunit A and B shows that their *N*-terminal segments point in two distinct directions (residues 24–30 are shown in sticks, carbon atoms are color-coded according to the parent subunit). The maximal displacement of 19.75 (± 0.48) Å, is observed between Pro24 of two partner subunits (measured between their Cα atoms).

The structure of the HT-hTS R175C was solved to 2.25 Å resolution (Tables S1 and S2), showing the constitutive dimeric quaternary structure of the enzyme (Figure 2a). The two homodimers (A-B and C-D, in our model) found in the cell asymmetric unit (ASU, Figure 2a) were fully traced, apart for the first 22–23 *N*-terminal residues (further the 12 residues belonging to the non-removable His^6^-tag) and the C-terminal valine. Within each enzyme homodimer, both subunits adopt the active conformation, showing the catalytic Cys195 exposed inside the active site cavity (Figure 2a). In all subunits, Cys195 results oxidized to S-oxy-cysteine (CSX195, Figure 2a). The two dimer halves are almost identical, apart for their *N*-terminal segments that point in two distinct directions as visible in the structural comparison displayed in Figure 2b. The maximal displacement, 19.75 (± 0.48) Å, is observed on Pro24 (measured between their Cα atoms). 

**Figure 3:**
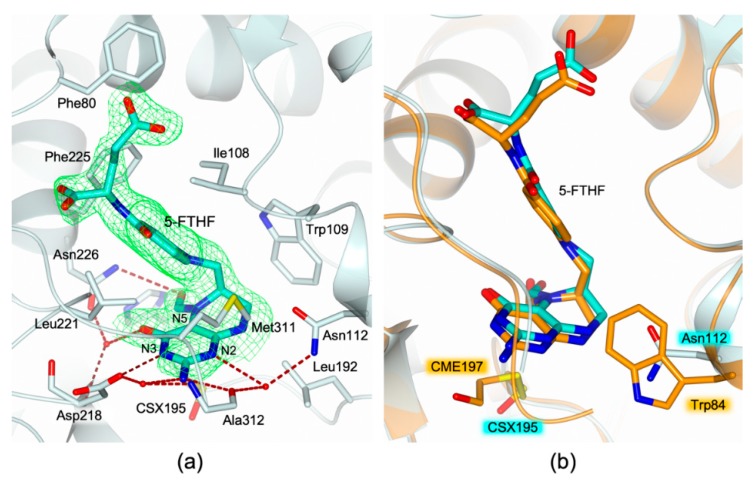
(**a**) Active site view of the B subunit of HT-hTS R175C (light cyan cartoon and carbon atoms). The catalytic Cys195 is modified as S-oxy-cysteine (CSX195, in sticks). The cofactor analogue 5-formyl-6-tetrahydrofolate (5-FTHF, in sticks, cyan carbons) is entrapped within the enzyme active site by a tight network of H-bonds (tan dashed lines) and van der Waals interactions. The ligand is surrounded by the omit map contoured at the 3σ level. (**b**) Active site view of the superimposition between the structures of HT-hTS R175C (light cyan cartoon and carbon atoms) and the bacterial *Enterococcus faecalis* TS (*Ef*TS, orange cartoon and carbon atoms; PDB id 3UWL [18]). In both structures, the active site of the enzyme is populated by the cofactor analogue 5-FTHF (in sticks, carbons are colored cyan and orange in HT-hTS R175C and *Ef*TS, respectively). The binding mode of the ligand is conserved in both complexes. The catalytic cysteine is modified as S-oxy-cysteine (CSX195, in sticks) in the structure of HT-hTS R175C, and as S,S-(2-hydroxyethyl)thiocysteine (CME197, in sticks) in *Ef*TS.

Both enzyme dimers display asymmetrical ligand binding within their active sites (Figure 2a), a yet unreported phenomenon for hTS [15,16]. Indeed, an extra-electron density, whose shape is highly compatible with a folate-like molecule, is observed in only one catalytic cavity of each dimer (Figure 3a). The refinement demonstrated that the ligand is a tetrahydrofolate (THF) derivative since the pyrazine ring of the folate pteridine moiety was in a bent conformation peculiar of the cofactor reduced form (Figure 3a). The substituent on the pteridine C5 was a bi-atomic species, consistent with either an hydroxymethyl moiety, 5-hydroxymethyl-6-tetrahydrofolate (5-HMTHF), or a formyl group, 5-formyl-6-tetrahydrofolate (5-FTHF) (5-ethyl derivatives of the cofactor are not known). The ligand was refined as either 5-HMTHF or 5-FTHF, without meaningful changes in the refinement quality indicators and in the resulting Fourier maps, as expected. The resolution of the present structure (2.25 Å) did not allow distinguishing between single and double C-O bonds, preventing us to further speculate which folate-derivative populates this site. Nonetheless, we opted for the 5-formyl derivative because this molecule is naturally present inside cells [17] and it was formerly characterized in complex with another TS enzyme, the bacterial *Enterococcus faecalis* TS (*Ef*TS, PDB id 3UWL) [18]. 5-FTHF is stabilized into the cofactor binding site of HT-hTS R175C by a tight network of H-bonds and van der Waals interactions (Figure 3a) in analogous pose to that assumed in *Ef*TS (Figure 3b). The amine and ketone moieties of 5-FTHF pteridine ring form either direct or water mediated interactions with Asp218, Asn226, and Ala312. Furthermore, the carboxylate moiety of Asp218 is positioned only 2.81 (± 0.48) Å away from the pteridine nitrogen N3 strongly suggesting that N3 is protonated and donates a H-bond to the protein residue (Figure 3a). The formyl oxygen on the pteridine N5 takes a weak H-bond with the amide nitrogen of Asn226. The pteridine nitrogen N2 entails a water-mediated interaction with Asn112 and Ala312 (Figure 3a). Furthermore, 5-FTHF is stabilized in this site by van der Waals interactions with Ile108, Trp109, Leu192, Leu221, Phe225, and Met311. The majority of these interactions are conserved also in the structure *Ef*TS [18]. The catalytic cavities of bacterial and human enzymes are remarkably similar, apart for the hTS residue Asn112 that is replaced in by Trp84 in *Ef*TS (Figure 3b). Nonetheless, both residues are involved in interactions with 5-FTHF. In HT-hTS R175C, Asn112 entails a water-mediated interaction with the ligand, whereas in *Ef*TS, Trp84 forms van der Waals interaction with it. 

**Figure 4:**
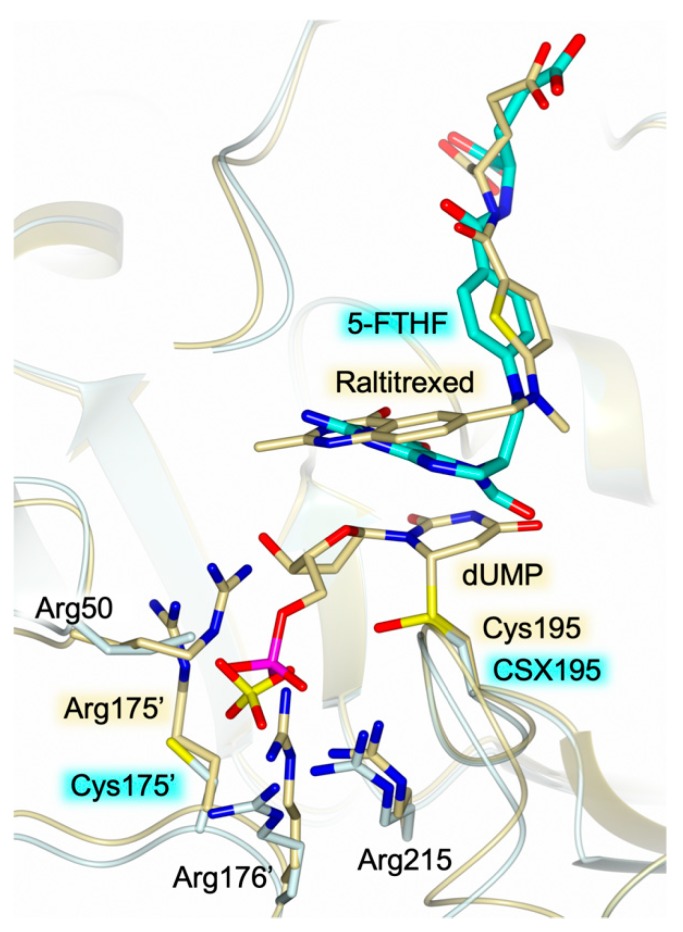
Active site view of the superimposition between the structures of HT-hTS variant R175C (light cyan cartoon and carbon atoms, sulfate anion in sticks) in complex with 5-FTHF (in sticks, cyan carbons) and the wild-type hTS (gold cartoon and carbons) in complex with dUMP (in sticks) and raltitrexed (in sticks). The reduced pteridine moiety of 5-FTHF is rotated by ~28° with respect to the corresponding moiety of raltitrexed, protruding in the substrate uracil site. In the structure of HT-hTS R175C, the catalytic cysteine is modified as S-oxy-cysteine (CSX195, in sticks) while in the ternary complex hTS-dUMP-raltitrexed, Cys195 is covalently bound to dUMP.

The structure of HT-hTS R175C shows the population of the cofactor pocket independently from substrate binding. This is a quite uncommon feature in hTS since, to date, the structures reported for this enzyme in complex with cofactor analogue inhibitors have been determined only in presence of the substrate (ternary complexes) [5,11,19,20,21]. Our attempts to obtain crystals of HT-hTS R175C in complex with the substrate dUMP have been unsuccessful. The comparison with the structure of the ternary complex hTS-dUMP-raltitrexed (PDB id 1HVY [5] and PDB id 5X5Q [11]) demonstrates a different arrangement of 5-FTHF and raltitrexed within the cofactor site (Figure 4). Indeed, the reduced pteridine moiety of 5-FTHF is rotated by ~28° with respect to the bicyclic system of raltitrexed, protruding in the substrate uracil site. This finding suggests the potential development of inhibitors able to concomitantly cover both the substrate and cofactor sites. 

**Figure 5:**
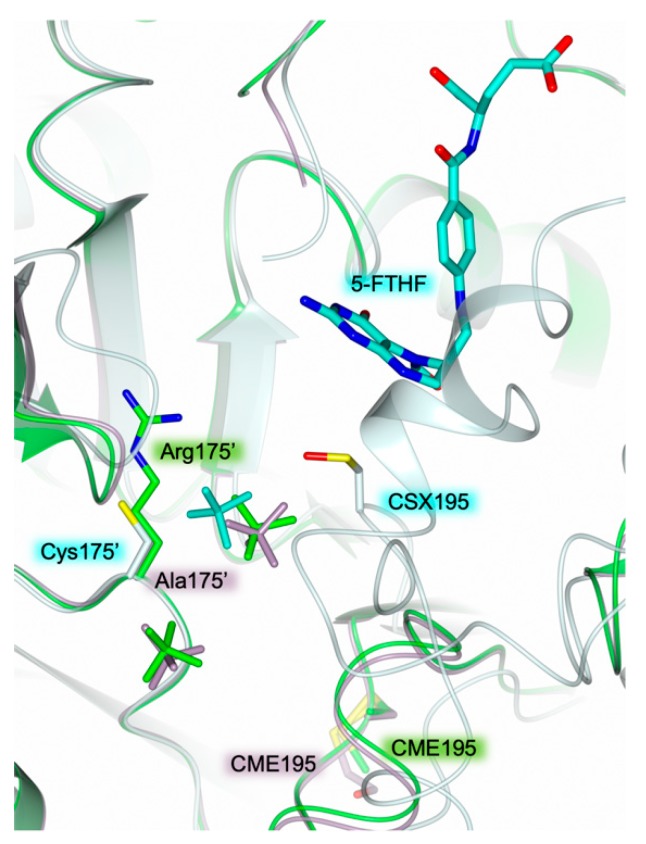
Active site view of the superimposition between the structures of HT-hTS R175C (light cyan cartoon and carbon atoms; 5-FTHF, in sticks, cyan carbons), the wild-type hTS (green cartoon and carbons; PDB id 3N5G [12]), and the hTS variant R175A (lilac carton and carbons; PDB id 4KPW [9]). The wild-type hTS and the R175A variant are in the inactive conformation, showing the catalytic Cys195, modified as S,S-(2-hydroxyethyl)thiocysteine (CME195, in sticks; in the structure of the wild-type enzyme the terminal oxygen and carbon atoms of CME195 are not visible), exposed at the dimer interface. On the other hand, HT-hTS R175C is in the active conformation showing the catalytic residue, modified as S-oxy-cysteine (CSX195, in sticks), exposed inside the active site. Sulfate ions (in sticks) are colored according to the parent molecules.

Structural investigations on hTS have shown a correlation between the enzyme active/inactive conformation and the ionic strength of the precipitant solutions used to crystallize the enzyme [4,5] (see Table S3). Low concentration of ammonium sulfate in the precipitant solution (below 0.2 M, low-salt condition) favours the switch of hTS to active conformation. Recent structural evidence on hTS has shown that the enzyme crystallizes in the active conformation under low-salt conditions regardless the presence of ligands bound to the active site (PDB id 4UP1) [22]. On the other hand, high concentration of ammonium sulfate in the crystallization solution (~ 1 M, high-salt condition) invariantly yielded the inactive conformation [5,12,23]. Presently, no structural characterization of hTS-substrate complexes has been obtained under high-salt conditions. Nonetheless, HT-hTS R175C displays the active conformation despite the high-salt condition (precipitant solution including 25 % saturated ammonium sulfate, corresponding to a concentration of ~ 1 M) applied to crystallize the protein. On the other hand, the structural characterization of the hTS variant R175A (PDB id 4KPW) yielded the enzyme in the inactive conformation under similar experimental conditions [9]. Notably, both the R175A and R175C variants are catalytically inactive [9]. The explanation of the quite unique structural behaviour of the R175C mutant is not obvious. Reasonably, in the R175C variant, the stabilization of the enzyme in the active conformation is due, at least in part, to the population of the cofactor site by 5-FTHF, not detected in the R175A variant. Since the mutated residue does not interact with the cofactor analogue, the complex formed in HT-hTS R175C is likely due to fortuitous conditions. On the other hand, in both variants, a sulfate anion (deriving from the crystallization environment) occupies the phosphate recognition pocket. The comparison with the structure of the R175A variant shows that the sulfate anion is moved by ~2.7 Å with respect to the position occupied in the R175C variant (Figure 5). In the R175A mutant, the sulfate matches the position of the same anion observed in the inactive conformation of the wild-type enzyme (Figure 5). In contrast, in the R175C variant, the position of the sulfate resembles that of the same anion in the structure of ligand-free hTS in the active conformation (PDB id 4UP1) [22]. This site almost matches the one occupied by the phosphate moiety of the substrate (Figure 4). The observed displacement, by ~1.2 Å, is reasonably due to the R175C mutation that removes one of the anchoring arginine of the cluster. Even though the variant is able to accommodate a sulfate anion in this site, its catalytic inefficiency indicates that dUMP binding is impaired, strongly suggesting that Arg175 is pivotal to anchor the substrate. 

## 3. Materials and Methods 

### 3.1. Macromolecule Production 

The expression plasmid for the hTS mutant R175C was a kind gift of Dr Hannu Myllykallio (Ecole Polytechnique, CNRS UMR7645, INSERM U696, 91128 Palaiseau, France). The variant was generated using the plasmid hTS-pQE80L (including the gene coding sequence for hTS cloned within the BamHI-HindIII restriction sites) as template, as already reported [9] (forward primer: AAGACGAACCCGGATGATTGCAGAATCATAATGTGTGCT; reverse primer: AGCACACATTATGATTCTGCAATCATCCGGGTTCGTCTT). 

The His^6^-tag hTS variant R175C (HT-hTS R175C, the non-cleavable *N*-terminal His^6^-tag was encoded by the pQE80L expression plasmid) was expressed in the *E. coli* strain BL21(DE3). Bacteria were cultured at 37 °C in the Luria Broath culture medium added by 100 mg L^−1^ ampicillin. Protein overexpression was induced when the OD_600nm_ reached the value of 0.6–0.8, by adding 0.4 mM isopropyl β-d-thiogalactopyranoside (IPTG). After 4 h, cells were harvested by centrifugation (5000 g, 15 min, 8 °C) and the resutling cell pellet was frozen at −20 °C (until required). Cells, resuspended in buffer A (50 mM Tris pH 6.9, 300 mM KCl), were lysed by sonication and the supernatant was subsequently separated by centrifugation (12000 g, 60 min, 8 °C). The target protein was purified according to an established procedure [9]. Briefly, the cell-free extract was applied to a HisTrap HP 5 mL column (GE Healthcare) and eluted using 250–500 mM imidazole concentration in the same buffer (step-gradient protocol). An almost pure (>95 %) protein sample was obtained after the first purification stage. Imidazole was removed through extensive dialysis in buffer A. The resulting sample was concentrated and further purified by size exclusion chromatography on a HiLoad 16/600 Superdex 75pg column (GE Healthcare). The elution profile was consistent with the enzyme dimer assembly (Figure S1). The high purity (>98 %) of the resulting protein sample was proven by SDS-PAGE analysis and MALDI-TOF mass spectrometry.

### 3.2. Kinetic Assays 

Enzyme activity assays were performed spectrophotometrically, according to a reported protocol [9]. Briefly, 1 mL reaction mixtures were prepared by adding aliquots of the enzyme (1–100 μg mL^−1^) to the assay buffer (50 mM TES, pH 7.4, 25 mM MgCl_2_, 6.5 mM HCHO, 1 mM EDTA, 75 mM β-mercaptoethanol) including variable concentrations of dUMP (5–200 μM) and mTHF (5–150 μM). Reactions, started by the addition of the substrate, were monitored by following the increase in absorbance at 340 nm during the oxidation reaction of mTHF to 7,8-dihydrofolate (DHF), for 3 min. 

### 3.3. Crystallization 

Dynamic light scattering (DLS) was performed on the purified HT-hTS R175C (0.54 μM, corresponding to 0.02 mg mL^−1^) in order to check the polydispersity of the protein in solution.

**Figure 6:**
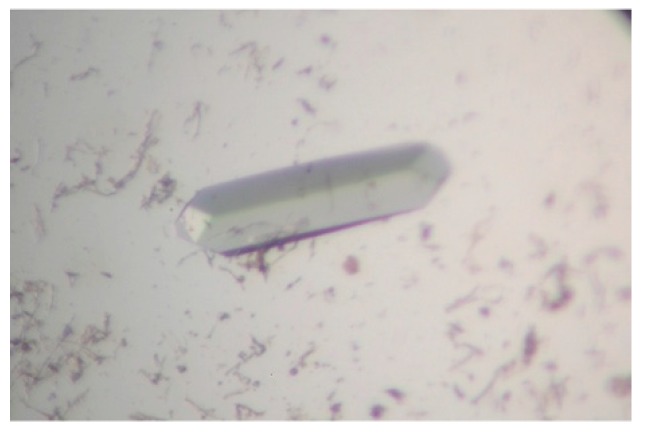
Crystals of the HT-hTS variant R175C.

Prior to the crystallization experiments the purified protein was extensively dialyzed in 0.1 M HEPES, pH 7.5 (at 8 °C) and then concentrated to 5 mg mL^−1^. The HT-hTS variant R175C was crystallized using the hanging drop vapour-diffusion method [24] at 20 °C. Drops were prepared by mixing equal volumes of protein (5 mg mL^−1^, in 0.1 M HEPES, pH 7.5, with or without 2 mM dUMP) and precipitant (25 % saturated ammonium sulfate, 20 mM β-mercaptoethanol, and 0.1 M TRIS, pH 8.3) solutions and equilibrated over 600 μL reservoir. Crystals (Figure 6), not isomorphous with those of the wild-type enzyme obtained under similar conditions, grew in 10–12 months. Before data collection crystals were washed in the cryoprotectant solution (20 % *v*/*v* ethylene glycol, 35 % saturated ammonium sulfate, 20 mM β-mercaptoethanol and 0.1 M TRIS, pH 8.3) and then flash frozen in liquid nitrogen.

### 3.4. Data Collection and Processing, Structure Solution and Refinement

X-ray crystallographic data were collected using synchrotron radiation at the European Synchrotron Radiation Facility (ESRF, Grenoble, France) beamline ID23-2, equipped with a MAR mar225 CCD detector. Reflections were indexed and integrated using the program XDS [25] and scaled with SCALA [26] from the CCP4 suite [27]. Crystals of HT-hTS R175C belonged to the primitive orthorhombic space group P2_1_2_1_2_1_, including four enzyme subunits (two enzyme dimers) in the cell asymmetric unit (ASU). Data collection and processing statistics are reported in Table S1. The structure of HT-hTS R175C was solved by molecular replacement using the software Molrep [28] from the CCP4 suite. One monomer of hTS in the active (PDB id 1HVY [5]) and inactive (PDB id 3N5G [12]) conformations were attempted as searching models (excluding water molecules and non-protein atoms), providing clear evidence that the enzyme crystallized in the active conformation (active conformation: score of 0.726 and wRfac of 0.418; inactive conformation: score of 0.604 and wRfac of 0.499). The model resulting from the molecular replacement using hTS in the active conformation (PDB id 1HVY [5]), was thus refined. The structure was refined with Refmac5 [29] from the CCP4 suite using the TLS parametrization [30] in the last cycles of refinement. The optimal partitioning of the polypeptide chains was calculated though the *TLS Motion Determination* web server [31], resulting in twenty continuous segments. The molecular graphic software Coot [32,33] was used for manual rebuilding and modelling of missing atoms. Water molecules were added through the ARP/wARP suite [34] and checked with Coot. Upon completion of the protein model, inspection of the Fourier difference map clearly evidenced the presence of a ligand bound within the active site of subunit B and D. The shape of the map indicated that the ligand was a derivative of tetrahydrofolate (THF) modified on the N5 of the pteridine ring. The two THF derivatives 5-formyl-6-tetrahydrofolate (5-FTHF) and 5-hydroxymethyl-6-tetrahydrofolate (5-HMTHF) were alternatively modelled and refined in this site. Furthermore, six sulfate anions from both crystallization and cryoprotectant solutions were found within the two enzyme dimers and hence included in the model. The occupancies of all exogenous ligands were singularly adjusted to values resulting in atomic displacement parameters close to those of neighboring protein atoms in fully occupied sites. The stereochemical quality of the final model was checked using Coot and Procheck [35]. Structure solution and refinement statistics are reported in Table S2. The model was rendered using the molecular-graphic software CCP4mg [36]. 

### 3.5. PDB Deposition

Atomic coordinates and structure factors for HT-hTS R175C were deposited in the Protein Data Bank under the accession codes 6QYQ.

## 4. Conclusions

Human TS (hTS) is considered an important target for anticancer chemotherapy. Nonetheless, the hTS-targeting drugs currently in use as anticancer agents, have limitations due to the onset of resistance. Thus, new strategies have to be explored to effectively target hTS without inducing resistance in cancer cells. Here, we report the structural and functional characterization of the novel hTS interface variant R175C. The mutation renders the enzyme catalytically inactive similarly to the previously reported R175A variant [9]. HT-hTS R175C crystallizes in the active conformation and shows subunit heterogeneity, as it entraps the cofactor analogue 5-FTHF within the catalytic cavity of only one subunit. 5-FTHF occupies the active site, adopting a conformation that partially hinders also the substrate pocket, suggesting the potential development of molecules able to concomitantly target both sites of the catalytic cavity. Further studies will address the understanding of the subtle factors that underlie the enzyme subunit heterogeneity, a phenomenon observed mainly in bacterial TSs [15,16,18,37]. The existence of half-site reactivity in TS enzymes is still a subject of investigation and debate [15,16,38,39]. On the other hand, the introduction of a cysteine residue at the dimer interface, as in the R175C variant, is exploitable for the development of innovative interface inhibitors through new drug discovery strategies based on the tethering approach [10]. This approach, relying on the formation of disulphide bonds between the enzyme and the ligand molecules, was successfully applied for the development of active site inhibitors of the bacterial *Escherichia coli* TS [10]. From this perspective, HT-hTS R175C is a functional tool to discover interface-targeting molecules. However, the activity of interface binders identified by tethering, should be tested on the wild-type enzyme. The application of interface-tethering on hTS is under investigation by our group. 

## Supplementary Materials

The following are available online. Figure S1: HT-hTS R175C elution profile during size exclusion chromatography, Table S1: Data collection and processing, Table S2: Structure solution and refinement, Table S3: Relationship between crystallization conditions and hTS conformational changes in the cited structures.

## References

[1] Houghton J.A., Houghton P.J. (1996). Cellular responses to antimetabolite anticancer agents: Cytostasis versus cytotoxicity. Prog. Cell Cycle Res..

[2] Chu E., Allegra C.J. (1996). The role of thymidylate synthase as an RNA binding protein. Bioessays.

[3] Liu J., Schmitz J.C., Lin X., Tai N., Yan W., Farrell M., Bailly M., Chen T., Chu E. (2002). Thymidylate synthase as a translational regulator of cellular gene expression. BBA Mol. Basis Dis..

[4] Schiffer C.A., Clifton I.J., Davisson V.J., Santi D.V., Stroud R.M. (1995). Crystal structure of human thymidylate synthase: A structural mechanism for guiding substrates into the active site. Biochemistry.

[5] Phan J., Koli S., Minor W., Dunlap R.B., Berger S.H., Lebioda L. (2001). Human thymidylate synthase is in the closed conformation when complexed with dUMP and raltitrexed, an antifolate drug. Biochemistry.

[6] Berger S.H., Berger F.G., Lebioda L. (2004). Effects of ligand binding and conformational switching on intracellular stability of human thymidylate synthase. BBA Proteins and Proteomics.

[7] Lin X., Liu J., Maley F., Chu E. (2003). Role of cysteine amino acid residues on the RNA binding activity of human thymidylate synthase. Nucleic Acids Res..

[8] Voeller D.M., Zajac-Kaye M., Fisher R.J., Allegra C.J. (2002). The identification of thymidylate synthase peptide domains located in the interface region that bind thymidylate synthase mRNA. Biochem. Biophys. Res. Commun..

[9] Salo-Ahen O.M.H., Tochowicz A., Pozzi C., Cardinale D., Ferrari S., Boum Y., Mangani S., Stroud R.M., Saxena P., Myllykallio H. (2015). Hotspots in an obligate homodimeric anticancer target. Structural and functional effects of interfacial mutations in human thymidylate synthase. J. Med. Chem..

[10] Erlanson D.A., Braisted A.C., Raphael D.R., Randal M., Stroud R.M., Gordon E.M., Wells J.A. (2000). Site-directed ligand discovery. PNAS.

[11] Chen D., Jansson A., Sim D., Larsson A., Nordlund P. (2017). Structural analyses of human thymidylate synthase reveal a site that may control conformational switching between active and inactive states. J. Biol. Chem..

[12] Cardinale D., Guaitoli G., Tondi D., Luciani R., Henrich S., Salo-Ahen O.M.H., Ferrari S., Marverti G., Guerrieri D., Ligabue A. (2011). Protein–protein interface-binding peptides inhibit the cancer therapy target human thymidylate synthase. PNAS.

[13] Pedersen-Lane J., Maley G.F., Chu E., Maley F. (1997). High-level expression of human thymidylate synthase. Protein Expres. Purif..

[14] Lovelace L.L., Gibson L.M. (2007). Lebioda cooperative inhibition of human thymidylate synthase by mixtures of active site binding and allosteric inhibitors. Biochemistry.

[15] Sapienza P.J., Falk B.T., Lee A.L. (2015). Bacterial thymidylate synthase binds two molecules of substrate and cofactor without cooperativity. J. Am. Chem. Soc..

[16] Finer-Moore J.S., Lee T.T., Stroud R.M. (2018). A single mutation traps a half-sites reactive enzyme in midstream, explaining asymmetry in hydride transfer. Biochemistry.

[17] Stover P., Schirch V. (1993). The metabolic role of leucovorin. Trends Biochem. Sci..

[18] Pozzi C., Ferrari S., Cortesi D., Luciani R., Stroud R.M., Catalano A., Costi M.P., Mangani S. (2012). The structure of Enterococcus faecalis thymidylate synthase provides clues about folate bacterial metabolism. Acta Cryst. D.

[19] Sayre P.H., Finer-Moore J.S., Fritz T.A., Biermann D., Gates S.B., MacKellar W.C., Patel V.F., Stroud R.M. (2001). Multi-targeted antifolates aimed at avoiding drug resistance form covalent closed inhibitory complexes with human and Escherichia coli thymidylate synthases. J. Mol. Biol..

[20] Almqvist H., Axelsson H., Jafari R., Dan C., Mateus A., Haraldsson M., Larsson A., Molina D.M., Artursson P., Lundbäck T. (2016). CETSA screening identifies known and novel thymidylate synthase inhibitors and slow intracellular activation of 5-fluorouracil. Nat. Commun..

[21] Almog R., Waddling C.A., Maley F., Maley G.F., Van Roey P. (2001). Crystal structure of a deletion mutant of human thymidylate synthase Δ (7–29) and its ternary complex with Tomudex and dUMP. Protein Sci..

[22] Deschamps P., Réty S., Bareille J., Leulliot N. (2017). Crystal structure of the active form of native human thymidylate synthase in the absence of bound substrates. Acta Crystallogr. Sect. F Struct. Biol. Cryst. Commun..

[23] Brunn N.D., Dibrov S.M., Kao M.B., Ghassemian M., Hermann T. (2014). Analysis of mRNA recognition by human thymidylate synthase. Biosci. Rep..

[24] Benvenuti M., Mangani S. (2007). Crystallization of soluble proteins in vapor diffusion for X-ray crystallography. Nat. Protoc..

[25] Kabsch W. (2010). XDS. Acta Crystallogr. D Biol. Crystallogr..

[26] Evans P. (2006). Scaling and assessment of data quality. Acta Crystallogr. D Biol. Crystallogr..

[27] Winn M.D., Ballard C.C., Cowtan K.D., Dodson E.J., Emsley P., Evans P.R., Keegan R.M., Krissinel E.B., Leslie A.G.W., McCoy A. (2011). Overview of the CCP4 suite and current developments. Acta Crystallogr. D Biol. Crystallogr..

[28] Vagin A., Teplyakov A. (2010). Molecular replacement with MOLREP. Acta Crystallogr. D Biol. Crystallogr..

[29] Murshudov G.N., Skubák P., Lebedev A.A., Pannu N.S., Steiner R.A., Nicholls R.A., Winn M.D., Long F., Vagin A.A. (2011). REFMAC5 for the refinement of macromolecular crystal structures. Acta Crystallogr. D Biol. Crystallogr..

[30] Winn M.D., Isupov M.N., Murshudov G.N. (2001). Use of TLS parameters to model anisotropic displacements in macromolecular refinement. Acta Crystallogr. Sect. D Biol. Crystallogr..

[31] Painter J., Merritt E.A. (2006). TLSMD web server for the generation of multi-group TLS models. J. Appl. Cryst..

[32] Emsley P., Cowtan K. (2004). Coot: Model-building tools for molecular graphics. Acta Crystallogr. D Biol. Crystallogr..

[33] Emsley P., Lohkamp B., Scott W.G., Cowtan K. (2010). Features and development of coot. Acta Crystallogr. D Biol. Crystallogr..

[34] Langer G., Cohen S.X., Lamzin V.S., Perrakis A. (2008). Automated macromolecular model building for X-ray crystallography using ARP/wARP version 7. Nat. Protoc..

[35] Laskowski R.A., MacArthur M.W., Thornton J.M. (1998). Validation of protein models derived from experiment. Curr. Opin. Struct. Biol..

[36] Potterton L., McNicholas S., Krissinel E., Gruber J., Cowtan K., Emsley P., Murshudov G.N., Cohen S., Perrakis A., Noble M. (2004). Developments in the CCP4 molecular-graphics project. Acta Crystallogr. D Biol. Crystallogr..

[37] Anderson A.C., O’Neil R.H., DeLano W.L., Stroud R.M. (1999). The structural mechanism for half-the-sites reactivity in an enzyme, thymidylate synthase, involves a relay of changes between subunits. Biochemistry.

[38] Wielgus-Kutrowska B., Grycuk T., Bzowska A. (2018). Part-of-the-sites binding and reactivity in the homooligomeric enzymes—facts and artifacts. Arch. Biochem. Biophys..

[39] Pozzi C., Ferrari S., Luciani R., Tassone G., Costi M.P., Mangani S. (2019). Structural comparison of enterococcus faecalis and human thymidylate synthase complexes with the substrate dUMP and its analogue FdUMP provides hints about enzyme conformational variabilities. Molecules.

