# Supplementary material for: Structural and Functional Characterization of the Human Thymidylate Synthase (hTS) Interface Variant R175C, New Perspectives for the Development of hTS Inhibitors
Source: Molecules. 2019 Apr 7;24(7):1362. doi: 10.3390/molecules24071362 (PMC6479699; doi:10.3390/molecules24071362)
Supplement: Supplementary file 1 [file molecules-24-01362-s001.pdf]

Article

Structural and Functional Characterization of the Human Thymidylate Synthase (hTS) Interface Variant R175C, New Perspectives for the Development of hTS Inhibitors

Cecilia Pozzi [1,*] Stefania Ferrari [2], Rosaria Luciani [2], Maria Paola Costi [2] and Stefano Mangani [1,*]

1 Department of Biotechnology, Chemistry and Pharmacy, Department of Excellence 2018-2020, University of Siena, via Aldo Moro 2, 53100 Siena, Italy; stefano.mangani@unisi.it

2 Department of Life Sciences, University of Modena and Reggio Emilia, Via Campi 103, 41125 Modena, Italy; sferrari591@gmail.com (S.F.); rosaria.luciani@gmail.com (R.L.); mariapaola.costi@unimore.it (M.P.C.)

* Correspondence: pozzi4@unisi.it (C.P.); stefano.mangani@unisi.it (S.M.); Tel.: +39-0577-232132 (C.P.); +39-0577-234255 or +39-0577-234252 (S.M.)

Table of contents:

Figure S1. S2
Table S1. S3
Table S2. S4
Table S3. S5
References. S5

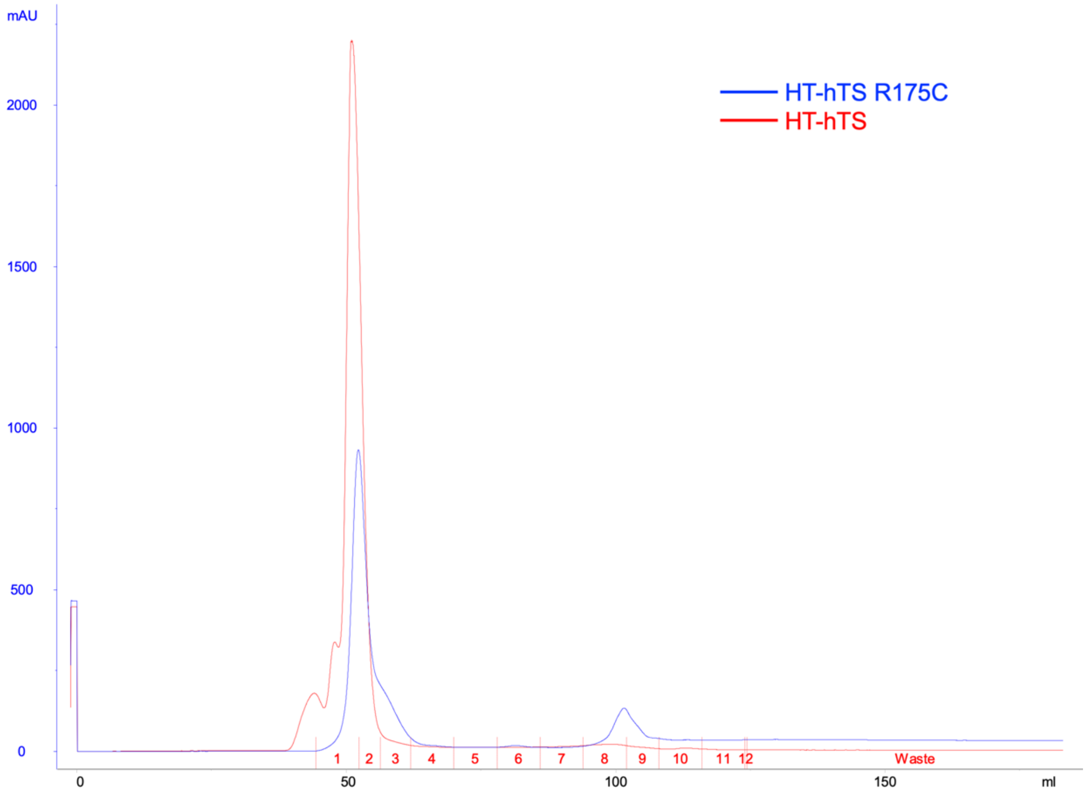

Figure S1. UV monitoring at 280 nm during size exclusion chromatography on a HiLoad 16/600 Superdex 75pg column (GE Healthcare). Elution profile of HT-hTS R175C (in blue) is overlaid with that recorded for HT-hTS (in red).

Table S1. Data collection and processing (Values for the outer shell are given in parentheses).

	**HT-hTS variant R175C**
**PDB code**	**6QYQ**
Diffraction source	ESRF ID23-2
Wavelength (Å)	0.8726
Temperature (K)	100
Detector	MAR mar225
Crystal-detector distance (mm)	224.4
Rotation range per image (°)	0.5
Total rotation range (°)	100
Exposure time per image (s)	1.5
Space group	$P2_12_12_1$
a, b, c (Å)	96.49; 96.52; 139.08
Mosaicity (°)	0.57
Resolution range (Å)	38.35-2.25 (2.37-2.25)
Total No. of reflections	212934 (30435)
No. of unique reflections	57746 (8475)
Completeness (%)	93.0 (94.7)
Redundancy	3.7 (3.6)
⟨I/σ(I)⟩	10.0 (3.8)
R_{meas}	0.085 (0.349)
Overall B factor from Wilson plot (Å^2)	39.99

Table S2. Structure solution and refinement (Values for the outer shell are given in parentheses).

	HT-hTS variant R175C
**PDB code**	**6QYQ**
Resolution range (Å)	36.70-2.25 (2.31-2.25)
Completeness (%)	92.53 (93.51)
No. of reflections, working set	54740 (4019)
No. of reflections, test set	2917 (229)
Final R_{cryst}	0.183 (0.492)
Final R_{free}	0.250 (0.533)
Estimated error on coordinated based on R value (Å)	0.337
No. of non-H atoms	
Protein	9146
Ion	34
Ligand	68
Water	476
Total	9724
R.m.s. deviations	
Bonds (Å)	0.011
Angles (°)	1.848
Average B factors (Å^2)	46.49
Ramachandran plot	
Most favoured (%)	96.1
Allowed (%)	3.9

Table S3. Relationship between crystallization conditions and hTS conformational changes, observed in crystal structures of wild-type and variants cited in this work.

PDB id	hTS type	Crystallization condition	Conformation
6QYQ	R175C variant (HT-hTS R175C)	High salt (25 % saturated ammonium sulphate, 20 mM β-mercaptoethanol, and 0.1 M TRIS, pH 8.3)	Active
4UP1 [22]	Wild-type	Low salt (25–30% PEG 4000, 30 mM ammonium sulfate, 20 mM β-mercaptoethanol, 100 mM Tris–HCl pH 9)	Active
4KPW [9]	R175A variant	High salt (30-40 % saturated ammonium sulphate, 20 mM β-mercaptoethanol, and 0.1 M TRIS, pH 8.8)	Inactive
3N5G [12]	Wild-type	High salt (30-40 % saturated ammonium sulphate, 20 mM β-mercaptoethanol, and 0.1 M TRIS, pH 8.8)	Inactive

References

9. Salo-Ahen, O.M.H.; Tochowicz, A.; Pozzi, C.; Cardinale, D.; Ferrari, S.; Boum, Y.; Mangani, S.; Stroud, R.M.; Saxena, P.; Myllykallio, H.; et al. Hotspots in an Obligate Homodimeric Anticancer Target. Structural and Functional Effects of Interfacial Mutations in Human Thymidylate Synthase. *J. Med. Chem.* **2015**, *58*, 3572–3581.

12. Cardinale, D.; Guaitoli, G.; Tondi, D.; Luciani, R.; Henrich, S.; Salo-Ahen, O.M.H.; Ferrari, S.; Marverti, G.; Guerrieri, D.; Ligabue, A.; et al. Protein–protein interface-binding peptides inhibit the cancer therapy target human thymidylate synthase. *PNAS* **2011**, *108*, E542–E549.

22. Deschamps, P.; Réty, S.; Bareille, J.; Leulliot, N. Crystal structure of the active form of native human thymidylate synthase in the absence of bound substrates. *Acta Crystallogr Sect F Struct Biol Cryst Commun* **2017**, *73*, 336–341.
